# Teacher Intervention During Collaborative Problem Solving in Mathematics Classrooms in Mainland China

**DOI:** 10.3390/bs15030377

**Published:** 2025-03-17

**Authors:** Yixuan Liu, Yiming Cao

**Affiliations:** 1 School of Mathematics and Statistics, Central China Normal University, Wuhan 430079, China; yxliuccnu@ccnu.edu.cn; 2 School of Mathematical Sciences, Beijing Normal University, Beijing 100875, China

**Keywords:** case study, collaborative problem solving, open-ended tasks, social activities, teacher intervention

## Abstract

In the Programme for International Student Assessment (PISA) 2015, students from four cities/provinces in mainland China performed worse in collaborative problem solving (CPS) than in other subjects. While student collaboration has been widely implemented in Chinese classrooms for over two decades, empirical research on teachers’ roles and interventions remains quite scarce. Influenced by international educational reform in the 21st century, educators have developed and made widespread use of open-ended tasks, perceived as more suitable for CPS, during mathematics lessons. In this study, we investigate the effect of teacher intervention during pair and small group CPS using a quasi-experiment with four teachers from eight classes. We then selected typical cases and analysed their effect on task performance regarding intervention focus and means. The result showed that three of the four teachers’ interventions proved effective. The most and least effective teachers were selected for the case study. We discuss teacher intervention’s effect in emphasising social activities and diagnosing. Considering the difference in authority between teachers in Chinese/Western classrooms, we discuss intervention styles and offer suggestions for choosing and carefully implementing appropriate forms of collaborative activities.

## 1. Introduction

In the current globalised world, collaboration and problem solving have received more attention from educational researchers, including learning and innovation skills within the 21st century skills framework (Bellanca, 2010). The Programme for International Student Assessment (PISA) 2015 measured collaborative problem solving (CPS) for the first time. Unlike other economies in the Asia-Pacific region, students from four cities/provinces in mainland China scored lower in CPS than predicted by their performance in the other three subjects (mathematics, reading, and science) (OECD, 2017b). Based on an analysis of PISA 2015 data, it is urged that CPS be permeated through all subjects (Tang et al., 2021).

In both PISA 2015 and ATC21S (Assessment and Teaching of 21st Century Skills), collaborative problem-solving competencies are deconstructed into collaboration (social skills) and problem solving (cognitive skills) (Griffin & Care, 2015; OECD, 2017a), both with a rich conceptual history. The effectiveness of collaborative/cooperative learning for achievement, attitudes, and perceptions has been widely proved through literature reviews (e.g., Bossert, 1988; Chen et al., 2018; Kyndt et al., 2013). It is now widely agreed that collaborative activities can better facilitate student learning, which has led to the global proliferation of collaborative learning in the classroom.

Collaboration is beneficial for students to develop a positive effect on mathematical problem solving and problem posing (Schindler & Bakker, 2020). Many activities/models in mathematics classrooms emphasise collaboration, for example, flipping classrooms (Q.-F. Yang et al., 2021), inquiry-based learning (Huang et al., 2021), and project-based learning (Bender, 2012). Internationally, collaborative learning and problem solving are both fostered within mathematics curricula in Western and Eastern countries such as the U.S. (Common Core State Standards Initiative, 2022), England (Department of Education, 2022), and Singapore (MOE, 2021). Research on mathematical problem solving has increasingly focused more on collaboration (Liljedahl & Cai, 2021). Some research considers collaboration as a part of the context for problem solving (Ng & Cui, 2021). In other cases, research is intended to better understand what collective problem solving entails (Sekiguchi, 2021; Zhang et al., 2022).

In mathematics classrooms, culture plays an integral role in the enactment of collaborative activities (Bryan et al., 2007; Lan et al., 2009). In East Asia, “[mathematics] classroom teaching is conducted in a whole class setting … there are virtually no group work or activities” (Leung, 2001). In 2001, the State Council of the People’s Republic of China (PRC) promulgated the Decision of the State Council on the Reform and Development of Basic Education, which encourages collaborative learning to promote mutual communication and development among students (State Council of the People’s Republic of China, 2001). Since then, collaborative learning has been actively promoted in classrooms for almost all subjects. As for Chinese mathematics curriculum standards mandated in 2011 and 2022, CPS and its components of collaboration and problem solving are highly emphasised, especially with the recent addition of project-based learning, which is highly rooted in and relies on CPS (Chan et al., 2018; Ministry of Education of the People’s Republic of China, 2011, 2022). Nevertheless, the reality of the implementation of collaborative learning in the classroom often became “a deliberately designed ‘show’ to meet the mandates” (cf. Wang & Wang, 2022), raising doubts about the effectiveness of teacher guidance in China.

Preparing appropriate collaborative tasks and dividing students into groups does not guarantee student collaboration. How teachers support students’ CPS activities is essential for effective learning outcomes since teachers are designers, organisers, and evaluators of collaborative activities (Davidson, 1990). Most research has found teacher intervention to have mostly positive effects on student learning outcomes (van Leeuwen & Janssen, 2019), but some negative results have been reported; for example, Ros (1993) found that more extended teacher contact was negatively correlated with students’ mutual elaboration. However, some research has found that the effectiveness of teacher intervention is more complicated than just positive and negative. Hogan et al. (1999), comparing situations with or without teacher guidance, found that teacher-guided discussions were more likely to attain a higher level of reasoning and explanations, noting that peer discussions without teacher guidance tended to be more generative and exploratory.

Although challenging, open-ended tasks with a low entry threshold but a high ceiling for potential achievement are more suitable for collaborative learning (Li et al., 2022). This conclusion is supported by research, indicating that open-ended problems promote collaborative learning and facilitate mathematical reasoning and communication (e.g., Kosyvas, 2016). In open-ended tasks, teachers need to relinquish some control over student activity so that students can build on their arguments rather than their teachers’ (Sullivan et al., 2015). Nieminen et al. (2022) illustrated that open-ended, real-life tasks may enable students to share agency in colloquial, non-mathematical discourses. However, Chan and Clarke (2017) argued that open-ended tasks might make participants’ learning activities less mathematically relevant, challenging teachers, noting that the effect of teacher intervention in open-ended tasks should be investigated.

In this paper, we analyse the current situation of Chinese teacher intervention in face-to-face CPS activities in mathematics classrooms to better understand the situation and promote more rational and scientific CPS activities. We first investigate whether teacher interventions affect students’ outcomes in open-ended tasks through a controlled quasi-experiment and then select typical cases of teacher intervention (positively and negatively effective cases) using coding frameworks of teacher intervention focus and means to discover the strengths and weaknesses of Chinese mathematics teachers when carrying out CPS activities.

## 2. Literature Review

### 2.1. Teacher Intervention During Collaborative Problem Solving

According to the recent research focusing on Chinese students by Zheng et al. (2023), both students’ internal and external perceptions can predict CPS competence in PISA 2015, through which teachers can affect students’ CPS performance. Through empirical research, it has been illustrated that CPS can be implemented in instruction and can be more effective than traditional instruction in students’ mathematical problem-solving performance (Anitha & Kavitha, 2023; Saadati & Felmer, 2021). Although there are successful examples of CPS included in the mathematics classroom, CPS is correlated with multiple domains (e.g., mathematics, sciences, reading, and environment; OECD, 2017a), it requires an appropriate theoretical framework to operationalise CPS into mathematics lessons. Subsequently, teacher intervention can be conceptualised based on previous literature reviews on the teacher’s role in collaborative activities.

**Figure 1:**
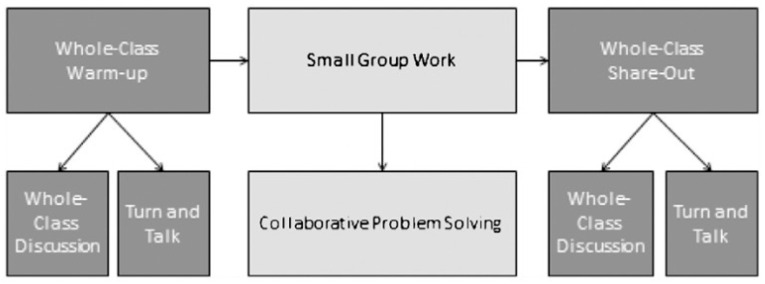
Participation structures posed by teachers in Webb et al. (2019).

First, CPS or collaborative learning should be conceptualised and situated in mathematics classrooms. Webb et al. (2019) found that teachers divided lessons into three phases: whole-class warm-up, small-group talk, and whole-class sharing. Collaborative problem solving is located within the small-group work phase (see Figure 1). Unlike individual and whole-class activities, we see CPS as a collaborative activity within the mathematics classroom focusing on mathematical problem solving.

**Figure 2:**
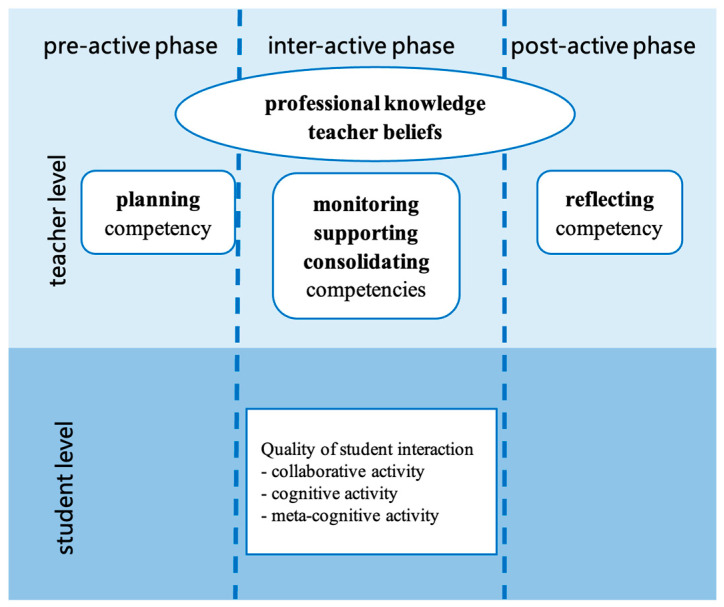
The ICLC framework by Kaendler et al. (2015).

The teacher’s role in collaborative activities is crucial across different aspects. Kaendler et al. (2015) compiled a framework, Implementing Collaborative Learning in the Classroom (ICLC), through a literature review. The framework clearly describes the teacher’s role in facilitating student collaboration and classifying teacher–student interactions at the student and teacher levels. The implementation of collaborative learning is divided into three stages—the pre-active phase (teacher’s lesson preparation and introduction of the collaborative learning setting before students’ work), the inter-active phase (students find solutions to the problem during the lesson), and the post-active phase (teacher reflects on the previous phases after the lesson)—with five different competencies across all three phases. As depicted in Figure 2, teacher competencies are based on the teachers’ professional knowledge and beliefs. Similarly, Webb (2009) classified the teacher’s role in student collaboration along several dimensions: preparing students for collaborative work, forming groups, structuring the group-work task, and influencing student interaction through discourse with small groups and the class.

To investigate the effectiveness of teacher intervention, we focus on the inter-active phase. By combining the teacher’s role in collaboration and the ICLC framework, we operationally define teacher intervention during CPS as a verbal intervention at the individual or pair/group level initiated by the students or teacher (excluding the language of non-task-related instructions) during pair and group collaboration. Because we focus on how teachers support student work, the teacher intervention defined here focuses only on monitoring and supporting during the inter-active phase of collaboration. We also omit discourse, such as reading out/explaining the task requirements and using directive language with the class.

### 2.2. Focus and Means of Teacher Intervention

As mentioned above, CPS competencies are divided into collaborative and problem-solving skills, a long-standing division in collaborative learning. Concerning teacher intervention research’s focus (intentions), focusing on students’ cognitive activities is distinguished from focusing on social activities in collaborative learning (e.g., Salmon, 2000). Molenaar et al. (2011) further distinguished between two levels of these activities: object and regulatory (meta). In an analysis of computer-supported collaborative learning, van Leeuwen et al. (2013) classified the focus of intervention along four dimensions with detailed descriptions, finding that the same teacher would intervene across lessons and groups using different foci. Teachers also tend to focus on students’ cognitive activities rather than social activities and intervene more often in groups with more student activities.

Through a review of the scaffolding of teacher–student interactions, van de Pol et al. (2010) found that some authors had begun to intentionally distinguish between the focus (what) and means (how) of scaffolding, arguing that such a division allowed researchers to observe teacher–student interactions more accurately. Their literature review summarised the most common intervention means: feedback, hinting, instructing, explaining, modelling, and questioning. van Leeuwen et al. (2013) added diagnosing and prompting to this list. According to the systematic review by van Leeuwen and Janssen (2019), almost all means of teacher intervention encouraged high-level cognitive processing and student engagement in the discussion. Feedback, prompting (hinting with questions), and questioning were more effective in facilitating student discussion.

Through the perspectives of focus and means of teacher intervention, the intentions and methods of the teacher can be illustrated. Research on scaffolding intentions in CPS can depict the current classroom situation through one lens and facilitate a development program for pre-service and in-service teachers (Haataja et al., 2019). The extent of teachers’ direct and indirect intervention and their level of control could be investigated by examining the specific methods of teacher intervention.

### 2.3. Teacher–Group Interactions in China’s Mathematics Classrooms

Previous mathematics classrooms in China were teacher-dominated, with minimal student involvement and hardly any group work (Leung, 2001). Collaborative learning was introduced much later in Chinese classrooms than in the West, initially posing many difficulties; for example, group learning was insufficiently developed (Wang & Wu, 2012), and teacher–group interactions were sorely lacking (Cao & He, 2009). Nevertheless, CPS has gained widespread popularity. In a 2013 survey of 13 Chinese provinces, 48.9% of teachers said they often carried out “collaborative learning in groups with teacher guidance”, while 60.3% said they often organised discussion activities for students (Shi et al., 2018), indicating a productive transformation from whole-class instruction to collaborative learning in mathematics classrooms.

However, some studies identify areas where teacher intervention should be reinforced. In some mathematics classrooms, teachers were more likely to prepare content for subsequent lessons or passively walk around the classroom when students worked in groups, interacting with students only when they found something wrong (Cao & He, 2009). In some classrooms, teachers mostly just “observed”, “nodded”, and “pointed” during their rounds, spending very little time in each student group (Wang & Wu, 2012). X. Yang et al. (2021) surmised that the experienced Chinese teachers in their study may have lacked knowledge of and experience with collaborative mathematics learning and could not identify students’ group-work weaknesses. Dong and Cao (2013) found that teachers focused more on cognitive assistance and that their guidance and evaluation of group communication were relatively lacking. The number of empirical studies on collaborative mathematics learning in China remains low (Wu et al., 2020), susceptive to the effectiveness, targets (focus), and methods (means) of teacher intervention.

### 2.4. Research Questions

This paper studies Chinese teacher intervention during students’ CPS on open-ended mathematics tasks. Although similar studies have been conducted in other countries and regions, research on secondary mathematics classrooms in China is relatively scarce. This paper addresses teacher intervention during face-to-face CPS in two types of collaboration—paired (two students) and small-group (four students)—often conducted in Chinese mathematics classrooms. Open-ended mathematics tasks aligned with the “ill-defined problems” applied in PISA are selected to optimise collaborative efficiency and encourage students’ participation. The research questions of this paper are as follows:

**RQ1.**  *How effective is teacher intervention in improving students’ performance during paired and small-group CPS in mathematics?*

**RQ2.**  *What is the potential relationship between the effectiveness of teacher intervention and the characteristics of teacher intervention (mainly focus and means) during student paired and small-group CPS in mathematics?*

## 3. Methodology

### 3.1. Participants

The participants are four mathematics teachers from two secondary schools in a developing district in Beijing. All four teachers had more than five years of teaching experience and had experience with and a positive attitude toward collaborative activities in their daily classrooms before the research. This study employed a quasi-experimental design, maintaining the original composition of the classes to preserve students’ mutual familiarity to establish a foundation for students’ collaboration. A total of 292 students in eight Grade 7 classes (12–13 years old) taught by four teachers were selected for the study. All four teachers claimed that both of their classes have similarities in motivation and attitude toward collaborative learning, and the differences in mathematics performance between the two classes led by the same teacher were not statistically significant (see Table 1). The classes taught by the same teacher were similar in size. Given the similarity in characteristics between the two classes taught by the same teacher, each teacher selected one of their two classes as the control class and the other as their intervention class according to their instructional schedule. In each class, students were instructed to perform three different open-ended tasks: individually, in pairs (two students each), and in small groups (four students each). Before the lesson commenced, teachers divided students into pairs and small groups, mostly based on their previous mathematics performances, to ensure that the average scores of all pairs and small groups were not significantly different. However, it is inevitable that while some pairs/small groups exhibited comparable mathematics performance, others demonstrated disparities in performance. The students were not told about the reason for grouping into pairs/small groups. Several pairs of three students and small groups of five to six students were formed in each class to involve all students. Each class had 16 to 20 pairs and 8 to 9 small groups. By controlling for task and student conditions, analysing task sheets and teachers’ behaviour could be helpful to explain the effect and enactment of teacher intervention.

The teachers were told to minimise intervention in the control classes and to be more involved in discussions and encourage students to collaborate effectively in the intervention classes. Teachers were also advised to encourage students to express their ideas to others in table or diagram form. Directly giving or evaluating the answer was forbidden because the task results would be attributed to the teachers rather than the students.

### 3.2. Tasks

This study considered two types of tasks: a paired task (two students for 12 min) and a small-group task (four students for 15 min). The CPS tasks used in the project were chosen from Clarke and Sullivan (Clarke, 1996; Clarke & Sullivan, 1990). The tasks were open-ended, meaning they had more than one correct solution and solution method. All the tasks were originally in English and were translated into Chinese by a native Chinese speaker fluent in English. Another person fluent in both languages then translated the Chinese tasks back into English. The research team then compared the differences between the original English tasks and the back-translated English tasks and discussed with the two bilingual translators the appropriate translation of the tasks.

Paired task

The average age of five people living in a house is 25. One of the five people is a Year 7 student.

What are the ages of the other four people and how are the five people in the house related? Write a paragraph explaining your answer.

(Chinese Translation)

一所房子有5个住户，他们的平均年龄是25岁，其中一位是七年级的学生。

其他四个人每人的平均年龄可能是多少岁？住在一起的这五个人有可能是什么关系？用一段话来解释你的答案。

Small-group task

Fred’s apartment has five rooms. The total area is 60 m^2^. Draw a plan of Fred’s apartment. Label each room, and show the dimensions (length and width) of all rooms.

(Chinese Translation)

小明的公寓有五个房间，而公寓的总面积是60平方米。

(1) 请画一幅图展示小明的公寓。
(2) 标注其中每一个房间的功能及尺寸（长和宽）。

### 3.3. Data Collection

Data were collected in the spring of 2017, beginning with data from the control classes, followed by data from the intervention classes. In each class, the students started by completing individual tasks (a warm-up for open-ended tasks) and then immediately collaboratively completing paired and then small-group tasks; students in the same pair were divided into the same group, which the process is the same as designed in Chan et al. (2018). After the lessons, the teachers were interviewed for their observations on student behaviour and performance. The data consisted of three components: classroom videos, task sheets, and video interviews with teachers.

### 3.4. Data Analysis

This study adopted a mixed-method approach combining quantitative and qualitative research methods. The quantitative analysis involved comparing the scores on the students’ task sheets in the control and intervention classes of the same teacher in the same task. The qualitative aspect of the study involved analysing the dialogues in the typical intervention classes.

The data analysis consisted of two parts: the scoring of students’ CPS sheets and the coding of teacher interventions by focus and means. In the process of problem solving, students in the same pair and small group filled in their results on one sheet, from which the CPS results were obtained. The scoring framework was piloted and polished to ensure that it clearly covered the performance of all students. The data to analyse the characteristics of teacher interventions were primarily derived from videos. The frameworks of teacher intervention based on focus and means were derived from previous research.

#### 3.4.1. Scoring of Task Sheet

The scoring criteria were based on paired (small-group) CPS tasks, with the score for each item indicating whether the student understood the relevant task content reasonably well and solved the problem. The scores for each item were summed to give an overall score for the paired (small-group) CPS result ranging from 0 to 5. Two coders scored the task sheets of all pairs and small groups independently. The consistency factors (Cohen’s Kappa) were 0.842 and 0.680, respectively, for the paired and small-group tasks, indicating interrater agreement at a medium level.

#### 3.4.2. Analysis of Teacher–Student Dialogues

The dialogue analysis units were closely related to this study’s research question. Since the focus was on teacher–student verbal interactions as the teacher moved among different groups within the classroom, the units of analysis were mostly divided at the turn level. One turn could sometimes contain more than one unit of discourse, and utterances were divided by “perceptible pauses”, commas, or full stops in the transcribed text (van Boxtel et al., 2000).

For the analysis of intervention focus and intervention means, the unit of analysis was each round of dialogue between the teacher and student. When synthesising van Leeuwen et al.’s (2013) coding of teacher intervention focus and means, each unit of analysis was coded as a unique intervention with focus and means, with the coding schemes in the Table 2 and Table 3. As shown in Table 3, the prompting description was based on hinting, so we combined them into the hinting category. All examples in Table 2 and Table 3 are from the current study. By analysing the focus and means of teacher intervention, a broad outline of teacher intervention styles can be further depicted.

## 4. Results

### 4.1. Task Sheets

As mathematics performance in the same teacher’s previous classes did not differ statistically, the CPS results can indicate the effectiveness of teacher intervention. As shown in Table 4 and Table 5, teachers T1, T3, and T4’s intervention classes outperformed their corresponding control classes in the paired task, while for teacher T2, the opposite held. In the small-group task, all intervention classes outperformed the corresponding control classes. It should be noted that students’ performance between the paired task and small-group task is not comparable because the topics and difficulties are different in the corresponding tasks.

### 4.2. Dialogue

The above discussion concerned the task sheet results, by which the intervention’s impact and effectiveness were judged. To further explore the impact of teacher interventions, T2’s and T4’s intervention classes were selected as cases for analysis. It seemed that T2’s intervention in paired tasks was not effective, since their performance was poorer than in the control class, while T4’s intervention seemed to be more effective than the corresponding control class. Both the focus and means of teacher intervention were further analysed.

**Figure 3:**
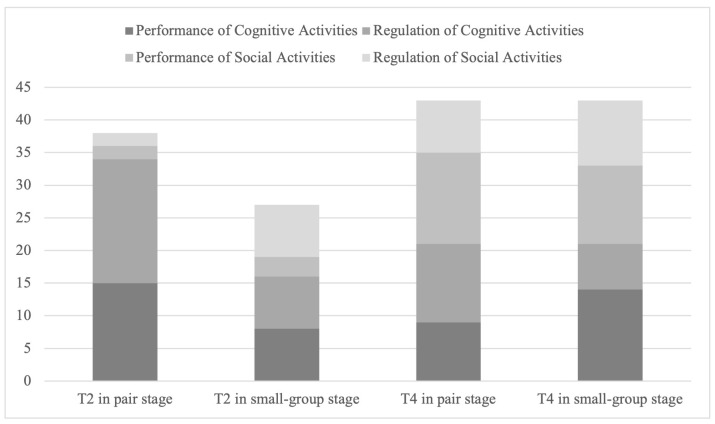
Focus of teacher intervention in different stages.

**Figure 4:**
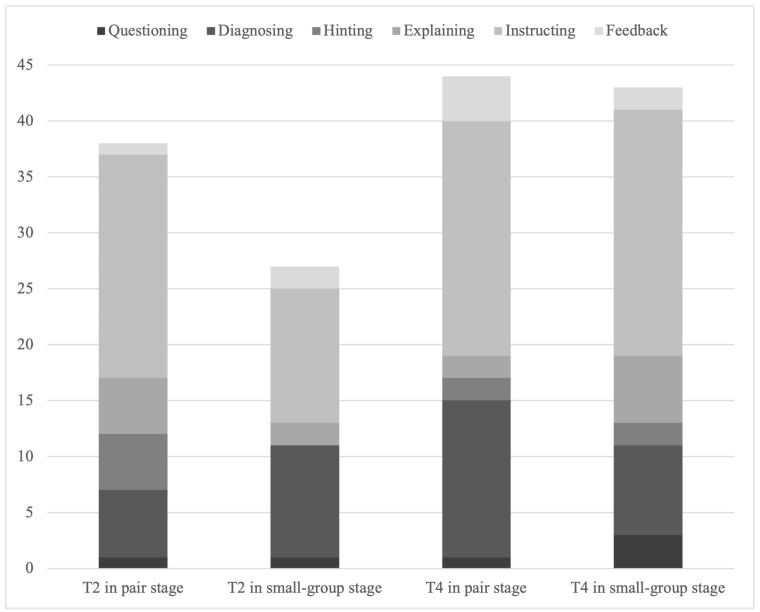
Means of teacher intervention in different stages.

T2 focused more on cognitive activities during interventions. During the paired stage, T2 initially focused more on cognitive performance and regulation, while with small groups, T2 indicated increased intervention for regulating social activities. Overall, T2 only made five interventions (7.69% of all T2’s interventions) for social activity performance in both stages. Interventions initiated by T2 are often commonly in the form of instructions (see Figure 3). During the paired stage, over half of T2’s intervention are in instructing form, while in the small-group stage, down to 44.44%. However, from the paired stage to the small-group stage, the proportion of diagnosing by T2 increased form 6/38 (15.79%) to 10/27 (37.04%) (see Figure 4).

T4’s inventions focused more equally among performance and regulation of cognitive and social activities. T4’s interventions focus was similarly distributed across the two stages (see Figure 3). As for means of T4’s interventions, instruction is the most common form, taking about half of all interventions. From the paired stage to the small-group stage, the proportion of explaining by T4 increased while the proportion of diagnosing decreased (see Figure 4).

## 5. Discussions

### 5.1. Social Activities and Diagnosing in Teacher Intervention

Overall, students in the intervention classes performed better than in the control classes in cognitive outcomes, except for T2’s students in paired work, which indicates that teacher intervention is generally effective (RQ1). Then, effectiveness is revealed from the teachers’ dialogue (RQ2). In the paired stage, T2’s interventions mostly focused on the cognitive level and less on diagnosing than T4. The ineffectiveness of T2’s interventions from the task sheet results may stem from such differences. When replying to students’ questions, T2 was more likely to encourage students to “think carefully”, while T4 encouraged students to “collaborate with others.” T4 mentioned words like “discussion” and “collaboration” (33/87) much more frequently than T2 (5/65). Further, “reasonable” was one of the most frequently mentioned words by T2 (12/65), much more frequently than by T4 (4/87). T4 also focused more on social activities in her diagnoses, always asking students about the results of their discussion, while T2 was more inclined to ask or observe the results of students’ writing on the task sheets.

Earlier research reports that cognitive and social activities significantly affect students’ collaborative learning activities in terms of performance and regulation level. Regulation of social activities (planning, monitoring, and evaluation of collaborative behaviours) significantly affects group performance (Janssen et al., 2012; Yager et al., 1986). As for social activities, while they can promote a positive atmosphere of collaboration among students, foster trust, and help group members to accomplish tasks more effectively (Wilson et al., 2006), too many social activities in group discussions may distract from the collaborative goals, impairing group performance (Janssen et al., 2012) and leading to negative socio-emotional behaviour that negatively impacts discussion (Webb et al., 2002). Comparable research has suggested that Chinese students may consider social-related dialogue implicit (Zhang et al., 2022), while relevant social guidance seems necessary. van Leeuwen and Janssen (2019) found a “mirror effect” between teachers’ focus on intervention and student collaborative activities. In other words, the dimensions of teacher intervention focus contributed to the corresponding student collaboration dimensions. Compared with T4, in the paired stage, T2 focused far more on cognitive aspects than social aspects, and more students tended to work independently than collaboratively, quite similar to the reason and result reported by Le et al. (2018), and less argumentations occurred in the pairs taught by T4, which may lead to less constructive dialogue between students (Cobb, 1995).

Increasing student participation is important for collaboration activities. Participating in conversations benefits students’ mathematics performance, particularly underperforming students (Webb et al., 2021). Teachers encouraging students to help each other facilitates greater student discussions through participation (Pijls & Dekker, 2011). Cognitive and social aspects both support and benefit students’ collaborative learning. Combining teachers eliciting student thinking and supporting student engagement with others’ ideas is potentially beneficial for student participation and student performance (Ing et al., 2015). In this study, T4 called on and encouraged several underperforming students to express their ideas individually and later followed up with diagnoses, while T2 did not. This difference may also explain the task sheet results. However, verbal diagnosis may be situationally more effective in facilitating student collaboration (Ros, 1993). Engeness and Edwards (2017) found that teachers’ checking students’ ideas allowed students to summarise and further explain their progress. In the current research, students checked by teachers preferred to explain their ideas to teachers for confirmation rather than other students in a group; in such a situation, T4 preferred to invite students to discuss their ideas with their group (social), while T2 preferred to encourage students to think more carefully (cognitive). This could also be the reason for the diversity between the classes. In the small-group stage, T2 changed his interventions to focus on social activities and diagnosing. Interestingly, his intervention class students outperformed his control class students in the small-group stage, possibly due to his refocusing. In sum, it is reasonable to deduct that the effectiveness of teacher intervention may come from social guidance and diagnosing (RQ2).

### 5.2. Intervention Style

Regarding the means of intervention, both teachers more frequently implemented high control-level interventions such as “explaining”, “instructing”, and “feedback”, while low control-level interventions such as “hinting” were less frequent, indicating that both teachers’ intervention styles exhibited high levels of control (Vermunt & Verloop, 1999).

Although the two teachers initiated similar intervention means in proportion, their intervention styles are different. T2 had a milder style than T4, who initiated more frequent interventions than T2. T4 was more prepared for intervention and would systematically target the silent students with direct interventions and discipline some students who talked off-task, while T2 did not. T2 always observed students’ performance with a smile and had fewer physical interactions with them, whereas T4 would call students by their names and put her hand on their shoulders when instructing them individually; this sort of extended hand-on-shoulder touching facilitated constructing a private dyadic conversation (Heinonen et al., 2020). When a teacher takes more control, students tend to depend more on them than on their groupmates (Ding et al., 2007). Such a situation was also found in the small-group stage: students in T4’s class asked more questions and expressed their views to the teacher more readily (to resolve disagreements) than those in T2’s class. Considering that on one hand, both teachers kept their intervention styles across the pair and group stages, while on the other hand, both teachers have effective intervention stages, it is hard to reveal the connection between intervention style and effectiveness (RQ2).

Previous research indicates that students in smaller groups are more involved in interactional activities than those in bigger groups (Lan et al., 2009; Ros, 1993). In the current study, both teachers noticed the silent pairs and encouraged collaboration. In the small-group stage, such encouragement was only given when half of the group members were observed as not collaborating or the group was still working in pairs; neither teacher intervened if only one student in a small group failed to participate. Considering that large groups may facilitate some students’ isolation and inactivity (Saqr et al., 2019), we suggest teachers arrange activities for students working in pairs or small groups based on cognitive demands and instructional objects to maximise each student’s opportunity to participate and possibly be more attended to by the teacher.

## 6. Conclusions and Limitations

In the current study, all participating teachers enacted the same tasks recommended by the authors, making it possible to investigate effectiveness of their interventions. Firstly, by analysing the task sheet results, we can conclude that teacher intervention was generally effective, except in one case at the paired stage. Secondly, this case was possibly attributable to the lack of intervention focusing on social activities and means of diagnosing, consistent with van Leeuwen and Janssen’s (2019) review. Previous research found that Chinese students spent less time on social considerations than Australian students, which may sometimes suggest miscommunication (Zhang et al., 2022). The current research may suggest the importance of social support from the teacher intervention perspective. Thirdly, although the two teachers in this case study had different intervention styles, their students were more successful in the pair (T4) or small-group (T2) task sheets than the corresponding control classes, indicating that both mild and strict intervention styles could be effective. Fourthly, teachers in the current study could more likely notice pairs without collaboration but more likely omitted silent individuals in groups. We suggest teachers arrange their collaborative activities in different group sizes depending on cognitive demands and the opportunity to be guided.

As instruction objectives are fixed on encouraging students to collaborate effectively, both teachers adhered to a “student-centred” principle in their interventions. As van Leeuwen and Janssen (2019, p. 86) noted, “[t]he challenge (in collaborative learning) is for teachers to remain central in supporting student learning, rather than controlling student learning opportunities”. In the current research, both teachers said they were surprised and happy to notice some underperforming students did well in participation. This could be attributed to the attributes of the open-ended questions (Li et al., 2022) and to the teacher’s control over providing assistance specifically related to the content (Pijls & Dekker, 2011).

This study has various limitations that should be discussed. Firstly, the control method majorly considers the similarity of students’ mathematics performance for the control and intervention classes and the environmental issue is only controlled through the teachers’ perspective. Secondly, the judgement of effectiveness and its analysis highly depend on the task sheet results, without sufficient input from the process of student discussion. Thirdly, because all students and teachers are videotaped in the whole lesson, the Hawthorne effect for the control and intervention classes may also influence the results and behaviour for teacher intervention. Fourthly, although all teachers had experience organising collaborative learning, they still felt challenged by the open-ended tasks in CPS, which, combined with the researchers’ suggestions, might influenced their behaviours in this study. Finally, the current study is a small study of a few classes, so we call for larger-scale studies within the varying cultural contexts for teacher intervention during students’ collaboration.

## Figures and Tables

**Table 1:** Previous mathematics performance of each class.

Teacher	Control Classes			Intervention Classes			Differences	
	N_1_	M_1_	SD_1_	N_2_	M_2_	SD_2_	M_2_–M_1_	*p*
T1	34	83.55	16.54	34	84.06	13.08	−0.51	0.888
T2	32	79.75	16.75	33	81.56	13.43	−1.81	0.635
T3	40	82.95	14.32	39	81.84	13.51	1.11	0.727
T4	39	79.14	16.68	41	77.65	19.28	1.49	0.720

**Table 2:** Coding scheme for the focus of intervention by van Leeuwen et al. (2013).

Focus	Descriptions	Examples
Performance of cognitive activities	Discourse about task content	“How do you calculate the size of the room?”
Regulation of cognitive activities	Discourse about planning of the task/time management Discourse about task strategies	“Read the problem again.” “Explain your answer.”
Performance of social activities	Discourse about the mood or other social activities within a group or the class	“Students should communicate well with each other.”
Regulation of social activities	Discourse about the collaboration process/about strategies for collaboration	“Distribute the tasks in the group.”

**Table 3:** Coding scheme for the means of intervention by van Leeuwen et al. (2013).

Means	Descriptions	Examples
Questioning	Request for a piece of information.	“How do you calculate the size of the room?”
Diagnosing	Questions to understand the current situation, without giving help. Asking what the problem is/about students’ understanding of the topic on hand.	“What is the question that you have not solved right now?”
Hinting	Giving a hint or a reminder without supplying the solution or detailed instructions. Students are still required to think for themselves. A hint can take the form of an instruction.	Student: “Is a balcony a room?” Teacher: “Is the area of the balcony counted as the area of your apartment?”
Explaining	Providing direct answers or information to elaborate on something, to make it clearer. After giving an explanation, the students(s) are able to continue their task immediately.	Student: “How big is a ten square meter bedroom?” Teacher: “It can probably fit a bed.”
Instructing	The teacher instructs students to do something. Recognisable mostly by the use of an imperative, but this is not necessary.	“Explain your answer.”
Feedback	Direct evaluation of the behaviour/work of the students.	“The state of your discussion is great.”
Other	Remaining utterances. Correcting a previous statement.	“That’s the last problem.”

**Table 4:** Mean and standard deviation performance of the paired task.

Teacher	T1	T2	T3	T4	Total
Control Class	3.94 (1.14)	4.28 (0.76)	4.20 (0.89)	3.76 (1.29)	4.04 (1.06)
Intervention Class	4.61 (0.54)	3.31 (1.83)	4.50 (0.68)	4.30 (0.75)	4.21 (1.15)

**Table 5:** Mean and standard deviation performance in the small-group task.

Teacher	T1	T2	T3	T4	Total
Control Class	2.81 (1.21)	2.81 (1.60)	3.11 (1.33)	2.94 (1.12)	2.93 (1.41)
Intervention Class	3.69 (1.20)	3.94 (1.37)	3.22 (0.73)	3.11 (1.41)	3.47 (1.30)

## Data Availability

The data are currently not publicly available due to participants’ privacy, but they are available from the first author upon reasonable request.
